# Vaccinomics-aided next-generation novel multi-epitope-based vaccine engineering against multidrug resistant *Shigella Sonnei*: Immunoinformatics and chemoinformatics approaches

**DOI:** 10.1371/journal.pone.0289773

**Published:** 2023-11-22

**Authors:** Sara Aiman, Abbas Ahmad, Asifullah Khan, Yasir Ali, Abdul Malik, Musaed Alkholief, Suhail Akhtar, Raham Sher Khan, Chunhua Li, Fazal Jalil, Yasir Ali

**Affiliations:** 1 Faculty of Environmental and Life Sciences, Beijing University of Technology, Beijing, China; 2 Department of Biotechnology, Abdul Wali Khan University Mardan, Khyber Pakhtunkhwa, Pakistan; 3 Department of Biochemistry, Abdul Wali Khan University Mardan, Mardan, Khyber Pakhtunkhwa, Pakistan; 4 National Center for Bioinformatics, Quaid-i-Azam University Islamabad, Islamabad, Pakistan; 5 Department of Pharmaceutics, College of Pharmacy, King Saud University, Riyadh, Saudi Arabia; 6 A.T. Still University of Health Sciences, Kirksville, Missouri, United States of America; 7 School of Biomedical Sciences, Chinese University of Hong Kong, Hong Kong, Hong Kong; The University of Kansas, UNITED STATES

## Abstract

*Shigella sonnei* is a gram-negative bacterium and is the primary cause of shigellosis in advanced countries. An exceptional rise in the prevalence of the disease has been reported in Asia, the Middle East, and Latin America. To date, no preventive vaccine is available against *S*. *sonnei* infections. This pathogen has shown resistances towards both first- and second-line antibiotics. Therefore, an effective broad spectrum vaccine development against shigellosis is indispensable. In the present study, vaccinomics-aided immunoinformatics strategies were pursued to identify potential vaccine candidates from the *S*. *sonnei* whole proteome data. Pathogen essential proteins that are non-homologous to human and human gut microbiome proteome set, are feasible candidates for this purpose. Three antigenic outer membrane proteins were prioritized to predict lead epitopes based on reverse vaccinology approach. Multi-epitope-based chimeric vaccines was designed using lead B- and T-cell epitopes combined with suitable linker and adjuvant peptide sequences to enhance immune responses against the designed vaccine. The SS-MEVC construct was prioritized based on multiple physicochemical, immunological properties, and immune-receptors docking scores. Immune simulation analysis predicted strong immunogenic response capability of the designed vaccine construct. The Molecular dynamic simulations analysis ensured stable molecular interactions of lead vaccine construct with the host receptors. *In silico* restriction and cloning analysis predicted feasible cloning capability of the SS-MEVC construct within the *E*. *coli* expression system. The proposed vaccine construct is predicted to be more safe, effective and capable of inducing robust immune responses against *S*. *sonnei* infections and may be worthy of examination via *in vitro*/*in vivo* assays.

## Introduction

*Shigella sonnei* (*S*. *sonnei*) is a rod-shaped, non-motile, gram-negative bacterium associated with actin polymerization in the host cell [1–5]. *S*. *sonnei* causes an acute intestinal infection known as shigellosis. *S*. *sonnei* originates from the harmless enterobacteriaceae family and causes debilitating diarrhea upon ingestion. The clinical symptoms of shigellosis can range from mild watery diarrhea to serious inflammatory bacillary dysentery with severe abdominal pains, fever, and bloody and mucus-filled stool [3]. Approximately 99% of shigellosis cases have been reported in underdeveloped countries with poor sanitation and hygienic conditions. Limited access to clear drinking water has encouraged the spread of enteric disease. Insufficient healthcare and malnutrition contribute to the high mortality rate in children, the elderly, and individuals with chronic health conditions [6]. Shigellosis is contagious and can easily be transmitted from person to person. Strong evidence links domestically acquired shigellosis associated with sexual transmission among homosexual men in Western industrialized countries as well as those with advanced HIV disease [6–8]. This bacterium spreads rapidly through contaminated food, water, or close oral contact with an infected individual.

Prevalence of shigellosis is increasing at an alarming rate in both developed and developing countries. Approximately 91 million instances of shigellosis occur every year with substantial morbidity and mortality worldwide [9]. Shigellosis ranked 3^rd^ in the United States [10] and ranked 6^th^ in China among gastrointestinal infections with high mortality rates [11]. In Asia, shigellosis alone causes 125 million infections each year and 14,000 fatalities. *S*. *sonnei* and *S*. *flexneri* accounted for approximately 90.5% of shigellosis cases [7, 12]. Rapid spread of shigellosis has also been reported in Europe, Asia, and Australia [6, 13, 14]. Typically, symptoms begin to appear 1–2 days after the infection and last for 7 days [8]. Shigella enterotoxin-1 and enterotoxin-2 (ShET-1 and ShET-2), the type 3 secretion system, and the invasion plasmid antigen H gene (IpaH) are main components contributing to the pathogenesis and survival of *S*. *sonnei* [15].

The majority of patients recover without antibiotic treatment; however, antibiotics administration is recommended to individuals with severe infections and underlying medical conditions that compromise the patient’s immune system [11]. Antibiotics are effective in preventing the spread of *Shigella* from one individual to another and in reducing the disease duration. However, recent studies have reported that *S*. *sonnei* develops resistance towards various antibiotics including trimethoprim, sulfamethoxazole, ampicillin, and fluoroquinolones [16]. Additionally, these strains have been reported to exhibit resistance to ciprofloxacin, a first-line treatment for shigellosis [17]. A variety of mechanisms, including drug extrusion by active efflux pumps [18], cellular permeability reduction, overexpression of enzymes responsible for inactivating and modifying drugs, and mutation-associated target modification, cause drug resistance in *S*. *sonnei* [19, 20]. The emergence of multi-drug-resistant *Shigella* strains and persistently high disease prevalence indicate that shigellosis is an ongoing and unresolved global health burden [21].

Currently, there is no commercial vaccine available against *Shigella*; however, a number of vaccines containing killed or live attenuated bacteria are being developed and evaluated at various clinical stages [3, 6]. The National Institute of Child Health and Human Development (NICHHD) and the Laboratory of Developmental and Molecular Immunology (LDMI) proposed a vaccine candidate, -O-SP- conjugated with a lipopolysaccharide (LPS) protein carrier against shigellosis to induce strong long-lasting immune responses; however, the induced immunogenic responses were found lower [22]. Therefore, the development of novel therapeutic strategies against multidrug-resistant *S*. *sonnei* is essential. Designing a potential vaccine against *S*. *sonnei* is an effective way to prevent this disease. The availability of mass genomic and proteomic data, and recent advancements in the fields of immunoinformatics and bioinformatics have greatly facilitated the design of safe and highly effective vaccines against particular pathogens [23]. In the present study, vaccinomics-based immunoinformatics strategies were employed to identify potential vaccine candidate proteins in *S*. *sonnei* proteome, followed by designing a multi-epitope-based vaccine construct using reverse vaccinology techniques. The vaccine was further evaluated for thermodynamic stability, binding potential to human immune receptors, and *in silico* cloning in a bacterial expression system. The efficacy of the designed vaccine construct was determined via immune simulation analysis by calculating the immunogenic responses in the host.

## Methodology

### Consent statement is not applicable for this study as there are no human participants or animal models involved in this study

**Fig 1:**
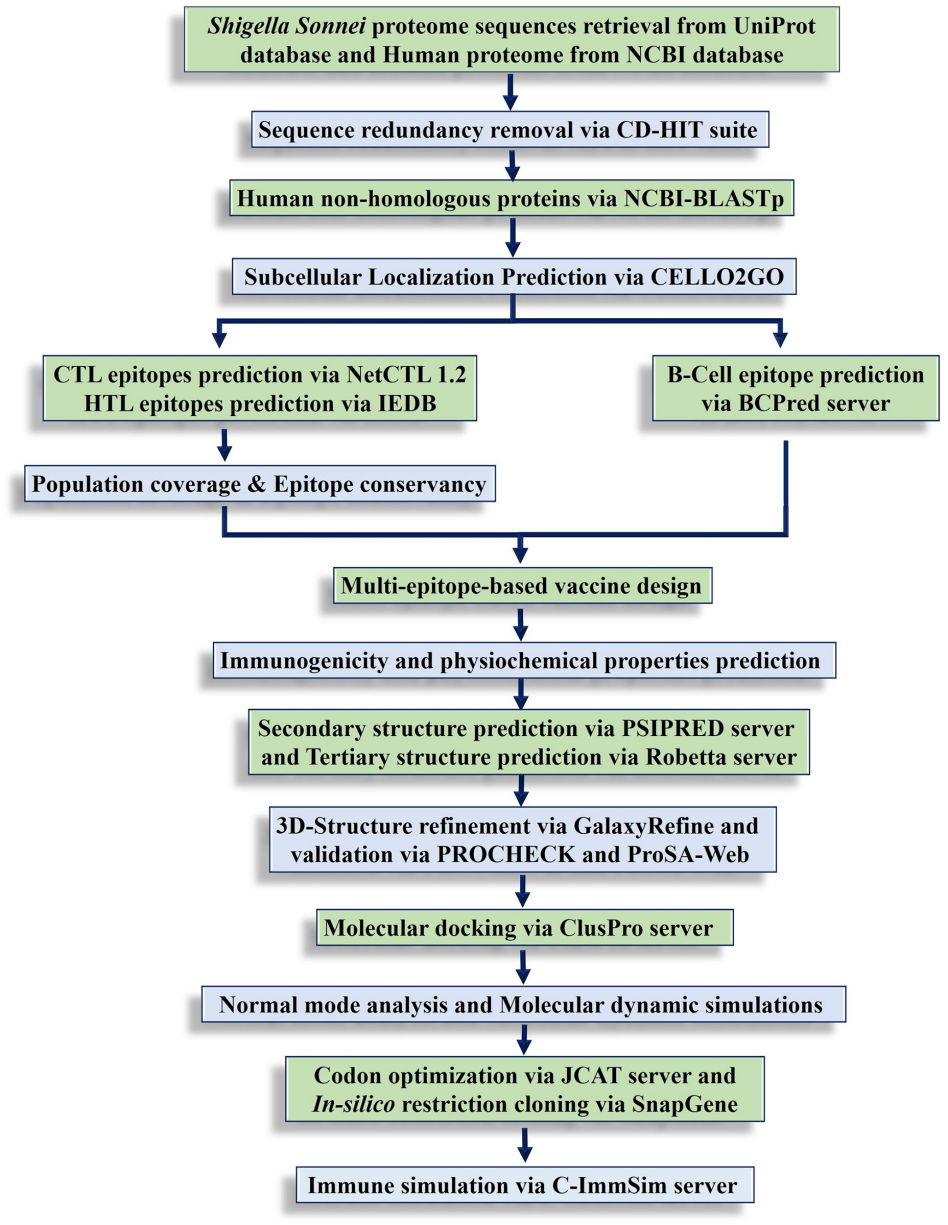
The systematic workflow of the current study.

Subtractive proteomic analysis was performed to identify pathogen-specific vaccine targets, and reverse vaccinology techniques were used to design a multi-epitope vaccine construct (MEVC) against *S*. *sonnei* (Fig 1).

### Proteome set retrieval and paralogous sequences identification

The entire proteome set of *S*. *sonnei* strains (available at the time this study was conducted) was retrieved from the UniProt database based on the UP000002529 accession ID (https://www.uniprot.org/, accessed on 30^th^ November, 2022) [24]. The data was subjected to the CD-HIT suite to identify paralogous protein sequences with a cut off value of 0.8 [25]. Redundant protein sequences were removed and non-paralogous protein sequences were acquired for further analysis.

### Human non-homologous proteins identification

Non-paralogous pathogen proteins were screened against the entire human proteome (Taxonomy ID: 9606) using the NCBI BLASTp program with a threshold of E-value cut off 10^−4^, percentage identity ≤35, query coverage ≤35, and bit score ≤100 [26]. Homologous proteins were removed, and pathogen-specific proteins were obtained during this subtractive analysis.

### Subcellular localization

Human non-homologous pathogen-specific proteins were subjected to subcellular localization using the CELLO2GO v.2.5 web server (http://cello.life.nctu.edu.tw/cello2go/, accessed on 3^rd^ December, 2022) during the downstream analysis. The server employs CELLO localization prediction and BLAST homology search approaches to screen for various properties of the targeted proteins. Additionally, the server provides data based on gene ontology (GO), including cellular components, molecular functions, and biological processes [27]. The outer membrane and extracellular proteins were prioritized as vaccine candidate proteins [28].

### Vaccine candidate proteins prioritization

VaxiJen v2.0 web server (http://www.ddg-pharmfac.net/vaxijen/VaxiJen/VaxiJen.html, accessed on 5^th^ December, 2022) was used to assess the antigenicity of the prioritized proteins with a threshold of >0.4 [29]. The Algpred2 web tool (https://webs.iiitd.edu.in/raghava/algpred2/, accessed on 5^th^ December, 2022) was used to evaluate the allergenicity of the prioritized antigenic proteins [30]. Non-allergenic antigenic proteins were further evaluated for toxicity using the ToxinPred2 server (https://webs.iiitd.edu.in/raghava/toxinpred2/, accessed on 5^th^ December, 2022) [31]. Subsequently, human host non-homologous pathogen proteins with antigenic, non-toxic, and non-allergenic natures were further analyzed for B- and T-cell epitope prediction.

### T-cell epitopes prediction

The MHC-I-restricted CD8+ cytotoxic T lymphocyte (CTL) epitopes in the prioritized pathogen proteins were predicted using NetCTL 1.2 web tool (https://services.healthtech.dtu.dk/services/NetCTL-1.2/, accessed on 7^th^ December, 2022) [32]. The epitope length setting maintained at 9-mers, and a maximum score of 0.75 was selected. The entire human HLA reference alleles set was used. The prioritized pathogen proteins were independently submitted in FASTA format, and the top two epitopes for each protein were prioritized based on antigenicity, allergenicity, and toxicity parameters [33]. MHC-II-restricted CD4+ helper T lymphocyte (HTL) epitopes were predicted for seven human HLA alleles (HLA-DRB1*03:01, HLA-DRB1*07:01, HLA-DRB1*15:01, HLA-DRB3*01:01, HLA-DRB3*02:02, HLA-DRB4*01:01, and HLA-DRB5*01:01) using the Immune Epitope Database (IEDB) MHC-II module (http://tools.iedb.org/mhcii/, accessed on 8^th^ December, 2022) [34]. The SMM-align (netMHC1.1) method was used to predict MHC-II epitopes, with a calling criterion of IC_50_ <500 nM. The IC_50_ values of HTL epitopes determined their affinity for MHC-II. The maximum binding affinity for MHC-II was observed at IC_50_ = 50 nM, moderate affinity for MHC-II was observed at IC_50_ value of 500 nM, and the lowest binding affinity for MHC-II was observed at an IC_50_ value of 5000 nM. In additional, epitopes were prioritized based on their antigenicity, allergenicity, and toxicity. The predicted epitope sequences with 20 mer were prioritized for further analyses. The conserved peptide sequences among various *S*. *Sonnei* strains were determined by the IEDB Epitope Conservancy tool (http://tools.iedb.org/conservancy/, accessed on 9^th^ December, 2022).

### B-cell epitopes prediction

The ABCpred web server (http://crdd.osdd.net/raghava/abcpred, accessed on 9^th^ December, 2022) was used to predict linear B-cell epitopes from prioritized pathogen proteins. B-cell epitopes are crucial for inducing humoral immune responses that stimulate B lymphocytes to produce antibodies against pathogens. ABCpred uses a Support Vector Machine (SVM) and artificial intelligence approaches to accurately predict the sequences of B-cell epitopes [35]. High-scoring B cell epitopes were prioritized based on their antigenicity, allergenicity, and toxicity parameters. The prioritized CTL, HTL, and B-cell epitopes were screened against human proteins via BLASTp with a threshold of E-values > 0.05. Epitopes above the threshold values were identified as human non-homologous peptides.

### Multi-epitope vaccine construct (MEVC) designing

Potential vaccine design requires an adjuvant, prioritized B- and T-cell epitopes, and linker sequences to design an efficient construct with the potential to elicit a robust immune response. Multi-epitope vaccine construct elicits a stronger immune response than the individual peptides. In this study, we used six CTL epitopes, nine HTL epitopes, and three linear B-cell epitopes with appropriate linkers to design a chimeric vaccine construct. The 50S ribosomal protein L7/L12 adjuvant was incorporated into the vaccine construct at the N-terminus to enhance its stability and induce both innate and adaptive immune responses. The rigid linker EAAAK was used to connect the adjuvant to the multi-epitope sequence. Glycine-proline-rich GPGPG linkers were used to conjugate linear B-cell and HTL epitopes. Furthermore, CTL epitopes were linked with flexible AAY linkers. These linkers provide stability, prevent self-folding in the vaccine construct, enhance immunogenicity, and improve defense mechanism against specific pathogens [36].

### Immunogenicity assessment of SS-MEVC

The designed vaccine construct was further subjected to immunogenicity analysis. The AlgPred server (https://webs.iiitd.edu.in/raghava/algpred2/, accessed on 13^th^ December, 2022) [30] was used to predict the allergenicity of the vaccine construct with an accuracy of 85% at a threshold of 0.4. The antigenic nature of the vaccine construct was determined by using Vaxijen v2.0 (http://www.ddg-pharmfac.net/vaxijen/VaxiJen/VaxiJen.html, accessed on 13^th^ December, 2022), with probable antigen calling criteria set at >0.4 and ANTIGENpro (https://scratch.proteomics.ics.uci.edu/, accessed on 13^th^ December, 2022) servers. Vaxijen v2.0 server calculates the antigenicity scores based on the physicochemical properties of the peptide vaccine sequence with an accuracy of 89% [37]. ANTIGENpro assesses antigenicity by performing a 10-fold-cross-validation of the peptide sequence against known datasets and identifying the protective aspects of the antigenic sequences [38].

### Physiochemical properties assessment of SS-MEVC

The ProtParam tool from ExPASy (https://web.expasy.org/protparam/, accessed on 17^th^ December, 2022) was used to predict various physicochemical properties of the designed vaccine construct. ProtParam was employed to calculate the amino acid content, molecular weight, stability, aliphatic indices, theoretical PI, solubility, and grand average of hydropathicity (GRAVY) of the vaccine construct [39].

### Secondary structure prediction

The PSIPRED 4.0 web tool (http://bioinf.cs.ucl.ac.uk/psipred/, accessed on 18^th^ December, 2022) was used to predict the secondary structure of the designed vaccine construct. The server is based on position-specific scoring matrices to predict transmembrane topology, transmembrane helices, and the recognition of fold and domain regions in the peptide sequence [40].

### Structure prediction, refinement and validation

The Robetta web tool (https://robetta.bakerlab.org/, accessed on 19^th^ December, 2022) was used to predict the tertiary structure of the vaccine construct. The server uses comparative modeling or de novo structure prediction methods to predict the five best three-dimensional (3D) models for a protein [41]. The top 3D model was selected and refined using the GalaxyRefine web tool (https://galaxy.seoklab.org/cgi-bin/submit.cgi?type=REFINE, accessed on 20^th^ December, 2022) [42]. The refined 3D-structure of the designed vaccine construct was further validated using the ERRAT, PROCHECK (https://saves.mbi.ucla.edu/, accessed on 21^st^ December, 2022) [43], and ProSA-Web (https://prosa.services.came.sbg.ac.at/prosa.php, accessed on 21^st^ December, 2022) [44] servers.

### Conformational B-cell epitopes prediction

The refined and validated 3D-structure of the designed vaccine construct was subjected to conformational B-cell epitope prediction using the ElliPro web tool (http://tools.iedb.org/ellipro/, accessed on 22^nd^ December, 2022). Linear B-cell epitope prediction is based solely on the amino acid sequence of the designed vaccine. However, some epitopes are also discontinuous or conformational, and rely on the 3D-structure of the antigenic vaccine instead of the linear sequence [45].

### Molecular docking of SS-MEVC with human immune receptor

The Cluspro web server (https://cluspro.org/login.php, accessed on 24^th^ December, 2022) was used for molecular docking of the vaccine construct with human toll-like receptor 2 and 4 (TLR2 and TLR4) to evaluate the molecular interaction of the designed vaccine construct with immunological receptor (Stony Brook, New York, USA). ClusPro server is a protein-protein docking tool which generates different models based on the binding energies [46]. The refined vaccine construct was docked against the TLR2 (PDB ID: 2Z7X) and TLR4 (PDB ID: 3FXI) homodimers. The docked complex of the designed vaccine and TLR4 with the lowest energy score was prioritized for further analysis. The PDBsum web tool (http://www.ebi.ac.uk/thornton-srv/databases/pdbsum/, accessed on 19^th^ December, 2022) was used to obtained information about the vaccine construct interaction with TLR4 [47].

### Normal modes analysis

The iMODS web server (https://imods.iqfr.csic.es/, accessed on 1^st^ January, 2023) was used for normal mode analysis (NMA) of the designed vaccine construct in complex with TLR4 to calculate the flexibility of the vaccine construct [48]. The server defines potential conformational changes, detects elastic network models, and resolutions using a variety of coarse-grained atomic representations, and provides an improved affine-model-based arrow representation of macromolecular complex domain dynamics. Based on NMA, the server calculates the structural dynamics of proteins and docked protein complexes with other proteins and ligands, and provides deformability, eigenvalues, variance, B-factor (mobility profiles), covariance maps, and elastic network data [49].

### Molecular dynamic (MD) simulations

The AMBER 20 package was used to perform molecular dynamic simulations for the designed vaccine construct and TLR4 docked complex. The initial system was prepared for the MD simulations using the Tleap module. The prioritized vaccine-receptor complex was solvated in TIP3P [50]. Hydrated cubic box with a boundary size of 10 Å. Subsequently, Na^+^ and CL^-^ ions were added to the system to neutralize the charge density. Additionally, during the preprocessing phase, energy minimization was carried out, including the 500-step energy minimization of hydrogen atoms, 1000-step energy minimization of water molecules with a 200 kcal/mol Å^2^ restraint on the remaining system, 1000-step energy minimization of all atoms with the exception of the 5 kcal/mol Å^2^ restraint on α-carbon atoms, and 300-step energy minimization of non-heavy atoms with 100 kcal/mol Å^2^. The systems were heated to 300 K using the NVT ensemble, maintaining the temperature constant, and hydrogen bond restriction was applied using Langevin dynamics [51] and the SHAKE algorithm [52]. The complex system was equilibrated for 1000 ps and compressed using the NPT ensemble, which limited the energy of the C atoms to 5 kcal/mol Å^2^. Finally, the CPPTRAJ module was used to conduct the production of the 100 ns simulation and to analyze the trajectory [53]. After the 100ns MD simulation was successfully completed, root-mean-square deviation (RMSD), root-mean-square fluctuation (RMSF), and radius of gyration (Rg) studies were performed to evaluate the strength of the vaccine-receptor protein interactions.

### Codon optimization and *in silico* restriction cloning

The Java Codon Adaptation Tool (JCAT) (http://www.jcat.de/, accessed on 3^rd^ January, 2023) was utilized for the reverse translation of the finalized vaccine sequence to cDNA and codon optimization to achieve maximum expression in the bacterial expression system after cloning [54]. JCAT evaluates the maximum possible expression potential of the cloned vaccine by calculating the codon adaptation index (CAI) and percentage of CG content. The optimum CAI value reported for favorable transcriptional and translational efficacy is 0.8–1 and the GC content is 30%-70% [28, 55]. The pET28a_TIAL1 (*E*. *coli* plasmid) for *in silico* restriction cloning was retrieved from the Addgene server [56]. The Snapgene tool (https://www.snapgene.com/, accessed on 3^rd^ January, 2023) was used for *in silico* restriction cloning of the optimized codon sequence in an *E*. *coli* expression system.

### Immune simulation

The C-ImmSim server (https://kraken.iac.rm.cnr.it/C-IMMSIM/, accessed on 6^th^ January, 2023) was used for computational immune simulation of the designed vaccine construct to evaluate the immunogenic potential of the designed vaccine [57]. The server uses various machine learning techniques to predict possible stimuli of the host immune system and provide information regarding cellular and humoral responses against antigens [58]. The standard clinical protocol recommends a four-week period between two vaccine doses [59]. In this study, we followed the protocol previously used by to carry out the immune simulation of the designed vaccine construct. The simulation parameters were set as default for time periods of 1h 84h and 168h. The human host leukocyte antigens HLA-A*0101, HLA-A*0201, HLA-B*0702, HLA-B*3901, HLA-DRB1*0101, and HLA-DRB1*0401 were selected for 1000 simulation steps.

## Results

### Subtractive proteomics analysis

In this study, we utilized a subtractive proteomic approach to identify pathogen-specific vaccine proteins and design a multi-epitope subunit vaccine. The whole proteome of *S*. *sonnei* was acquired from the UniProt database (UniProt ID: UP000002529) with a total of 4,068 proteins. CD-HIT resource was used to remove redundant protein sequences with a sequence similarity index of 80%. Non-paralogous pathogen proteins were further screened against the human proteome using BLASTp analysis to remove human homologous proteins. A total of 2633 human non-homologous and non-paralogous proteins from *S*. *sonnei* were acquired for downstream analyses (S1 File).

### Subcellular localization

CELLO2GO v.2.5 [27] categorized the prioritized list of proteins with respect to their cytoplasm, periplasm, inner membrane, outer membrane, or extracellular localization (Table 1) [25]. Three proteins with UniProt IDs Q3Z2A3, Q3Z118, and Q3YUS5 in the outer-membrane and extracellular region were prioritized based on higher antigenicity scores of 0.4611, 0.6350, and 0.6462, respectively (Table 2).

**Table 1:** Subcellular localization prediction of the shortlisted proteins.

S. No.	Cellular location	Number of Proteins
1.	Cytoplasm	1608
2.	Periplasm	358
3.	Inner membrane	496
4.	Outer membrane	99
5.	Extracellular	72

**Table 2:** The top-ranked vaccine candidate proteins with amino acid lengths, antigenicity, allergenicity, and toxicity.

No	UniProt ID	Name of protein	Number of Amino acids	Antigenicity	Allergenicity	Toxicity
1	Q3Z2A3	Sulfurtransferase	454	Antigenic	Non-allergen	Non-toxin
2	Q3Z118	Putative oxidoreductase	260	Antigenic	Non-allergen	Non-toxin
3	Q3YUS5	Single-stranded DNA-binding protein	182	Antigenic	Non-allergen	Non-toxin

### B- and T-cell epitopes prediction

The three proteins prioritized in this study were subjected to B- and T-cell epitope prediction to identify the lead epitopes for designing a potential MEVC against *S*. *sonnei*. T cell epitopes represent MHC class I and II molecules that are capable of stimulating CD8+ and CD4+ T cell receptors, respectively, to induce a strong immune response against specific pathogens [60]. NetCTL 1.2 tool predicted 17 CTL epitopes from the three proteins prioritized in this study (S1 Table). Only 6 epitopes were prioritized based on their antigenic, non-allergenic, and non-toxic nature, MHC binding scores, and non-homology to human host (Table 3). The IEDB database predicted a total of seven HTL epitopes of 15-mer based on an IC50 value of ≤500. Three HTL were further prioritized based on antigenic, non-allergenic, non-toxic, and human host non-homologous features (Table 4). The prioritized epitopes were conserved peptide sequences among various *S*. *Sonnei* strains and the epitope conservancy was calculated based on 100% sequence identity (Table 5). ABCpred identified 10 B-cell epitopes of the 20-mer with scores of >0.9. The three B-cell epitopes with the best scores were prioritized for incorporation into the vaccine construct to induce humoral responses against *S*. *sonnei* (Table 6).

**Table 3:** Top-ranked CTL epitopes prioritized for vaccine construct designing.

Uniprot IDs	Peptide sequence	Human non-homology (BLASTp, E-value >0.5)	MHC binding affinity	Rescale binding affinity	C-terminal cleavage affinity	Transport affinity	COMB score	MHC-I binding
Q3Z2A3	KADAPVALY	0.42	0.4936	2.9592	0.9752	2.9560	2.3899	Yes
	ISHIPGADY	9.8	0.2002	0.8499	0.8738	3.0660	1.1343	Yes
Q3Z118	KSDAGSLVF	57	0.3999	1.6977	0.9411	2.5540	1.9666	Yes
	LADEYQQRL	1.7	0.1368	0.5807	0.9674	0.8820	0.7699	Yes
Q3YUS5	KLAEVASEY	4.8	0.3174	1.3477	0.9733	3.0380	1.6456	Yes
	WTDQSGQDR	14	0.2222	0.9432	0.0652	1.3610	1.0210	Yes

**Table 4:** Top prioritized HTL epitopes shortlisted for vaccine construct designing.

Protein IDs	Allele	Human non-homology (BLASTp, E-value >0.5)	Peptide sequence	Methods	Percentile rank
Q3Z2A3	HLA-DRB1*03:01	1.3	AKPLTLDQLQQQNGK	Consensus (smm/nn/sturniolo)	1.3
	HLA-DRB3*01:01	11	CGTGWRASETFMYAR	Consensus (comb.lib/smm/nn)	2.5
	HLA-DRB1*03:01	1.3	QIMLYAGVKDVRLLD	Consensus (comb.lib/smm/nn)	2.8
Q3Z118	HLA-DRB3*01:01	11	LPLLLKSDAGSLVFT	Consensus (comb.lib/smm/nn)	1.3
	HLA-DRB3*02:02	2.7	QDVMQVNVNATFMLT	NetMHCIIpan	1.4
	HLA-DRB1*03:01	3.8	MMQVLADEYQQRLRV	Consensus (smm/nn/sturniolo)	1.6
Q3YUS5	HLA-DRB3*02:02	11	PEVRYMPNGGAVANI	NetMHCIIpan	1.2
	HLA-DRB1*15:01	1.9	GKLAEVASEYLRKGS	Consensus (smm/nn/sturniolo)	1.1
	HLA-DRB3*02:02	5.4	VVVNVGGTMQMLGGR	NetMHCIIpan	1.3

**Table 5:** T-cell epitopes conservancy prediction by IEDB prioritized for vaccine construct designing.

Uniprot IDs	CTL Peptides	Epitope Conservancy	HTL Peptides	Epitope Conservancy
Q3Z2A3	KADAPVALY	100%	AKPLTLDQLQQQNGK	100%
	ISHIPGADY	100%	CGTGWRASETFMYAR	100%
Q3Z118	KSDAGSLVF	100%	QIMLYAGVKDVRLLD	100%
	LADEYQQRL	100%	LPLLLKSDAGSLVFT	100%
Q3YUS5	KLAEVASEY	100%	QDVMQVNVNATFMLT	100%
	WTDQSGQDR	100%	MMQVLADEYQQRLRV	100%

**Table 6:** The predicted B-cell epitopes prioritized for the vaccine construct designing.

Protein IDs	Epitopes	Peptide sequence	Human non-homology (BLASTp, E-value >0.5)	Position	Amino acid length	ABCpred Score
Q3Z2A3	B-cell	DYIDTNEVESEPLWNKVSDE	16	195	20	0.89
Q3Z118	B-cell	VNATFMLTQALLPLLLKSDA	5.7	125	20	0.86
Q3YUS5	B-cell	GGAQSRPQQSAPAAPSNEPP	1.4	150	20	0.96

### Model vaccine construction

**Fig 2:**
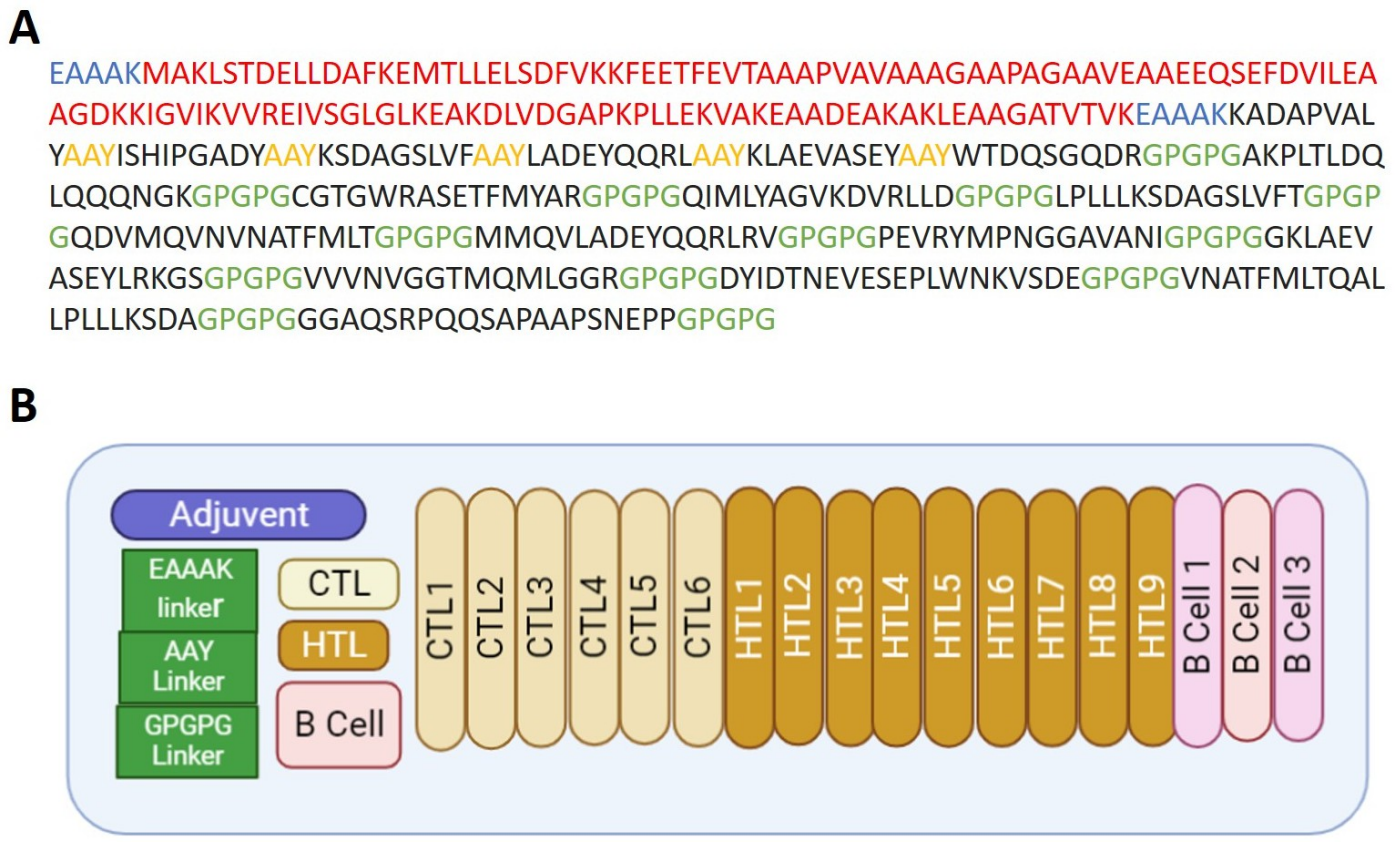
(A) Sequence of SS-MEVC design showing adjuvant in red, epitopes in black, and linkers in blue, yellow, and green. (B) Structural representation of the vaccine construct in different colors.

A multi-epitope chimeric vaccine construct was designed using prioritized CTL, HTL, and B-cell epitopes with the aim of eliciting a strong immune response against *S*. *sonnei* infections. Highly immunogenic vaccine design requires the presence of an adjuvant, antigenic epitopes, and appropriate epitopes-specific linkers. The L7/L12 ribosomal protein was incorporated as an N-terminus-linked adjuvant to increase the potency and minimize the toxicity of the designed vaccine. A total of six CTL, nine HTL, and three B-cell epitopes were used in the construct design. GPGPG linkers were used to link HTL epitopes with B-cell epitopes, CTL epitopes were linked with AAY linkers, and EAAAK linker was used to conjugate adjuvant to the epitopes. These epitope-specific linkers are critical in designing an immunogenic multi-epitope vaccine for the epitopes to elicit immune responses independently and as a whole construct without interfering with one another. The length of the model *S*. *sonnei* vaccine construct (SS-MEVC) was 469 amino acids (Fig 2). The proposed SS-MEVC vaccine is a superior alternative because it is inexpensive, easy to produce, stimulates specific immune responses, and reduces the risk of antigen-induced anaphylaxis. The designed vaccine construct could accommodate new or different antigenic peptide sequences.

### Immunological and physiological properties of SS-MEVC

The Protparam server was used to determine a number of different physicochemical parameters to confirm the stability of SS-MEVC. The server revealed that the vaccine construct had a theoretical PI value of 8.56, indicating slight acidic nature of the construct. The molecular weight of the vaccine construct was 39.18 kDa, suggesting its easy purification. The aliphatic index and GRAVY of SS-MEVC were 72.77 and -0.198, respectively. The instability index score of 37.04 indicated the stable nature of the vaccine construct. In yeast and *E*. *coli* expression systems, the half-life of the vaccine sequence was predicted as >20h and 10h, respectively [39]. Furthermore, the SS-MEVC construct was predicted to exhibit high antigenic, non-allergenic, and non-toxic behaviors. The ANTIGENpro antigenicity score of 0.907043 and VaxiJen v2.0 score of 0.7648 indicate the strong antigenic nature of SS-MEVC. SOLpred score of 0.939657 indicated high solubility of SS-MEVC upon expression. The immunological and physicochemical properties confirmed the non-allergenic, and non-toxic nature of the SS-MEVC construct along with stability and substantial capability to induce robust immune responses against *S*. *sonnei* infections.

### Secondary and tertiary structure prediction and validation

**Fig 3:**
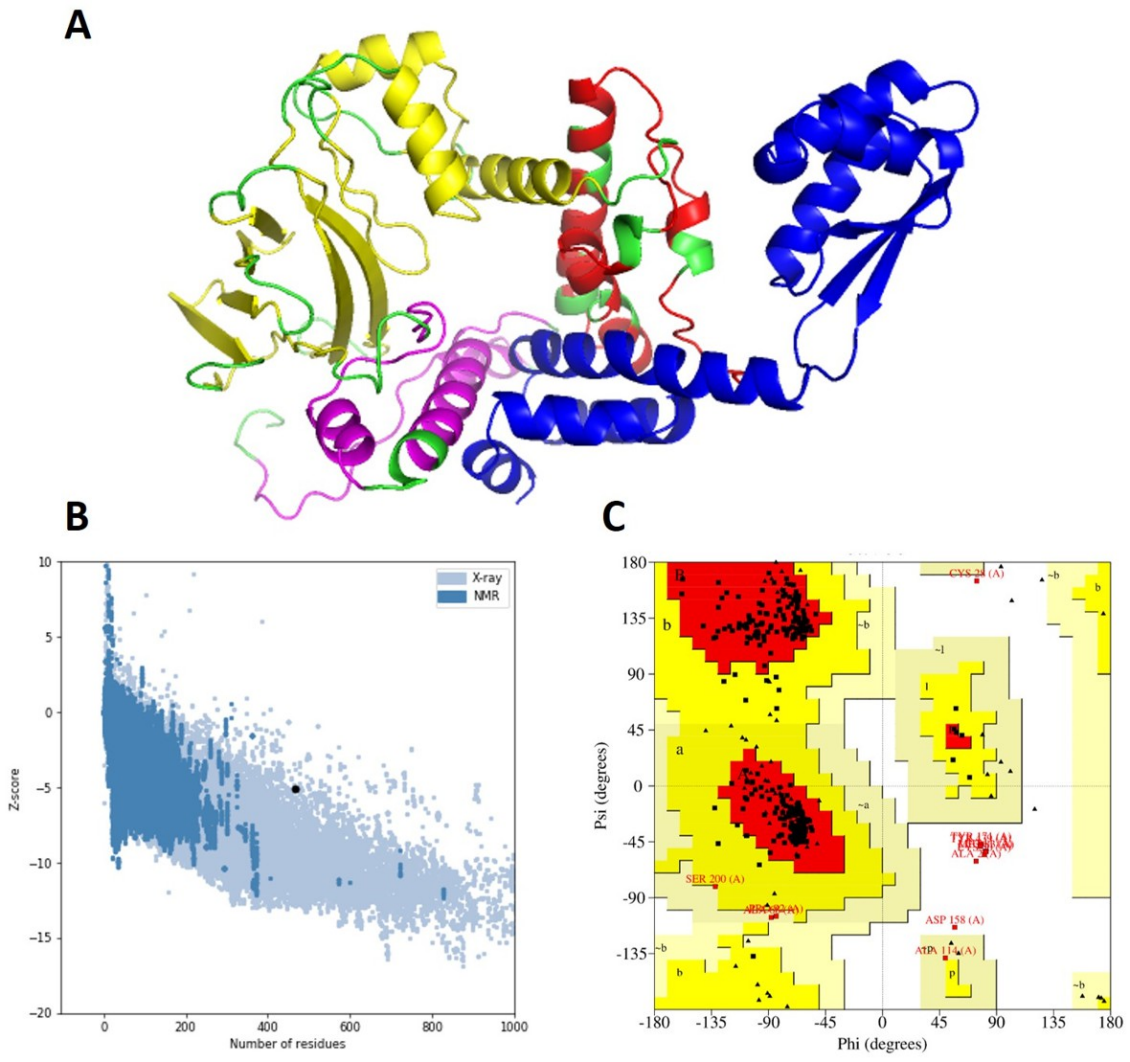
Tertiary structure prediction and validation of SS-MEVC. (A) The three-dimensional structure of the vaccine modeled by Robetta server. Blue, yellow, green, red, and purple represent the adjuvant, CTLs, HTLs, B-cell epitopes, and linkers, respectively. (B) Structure validation of SS-MEVC by ProSA-web with a Z-score of -4.78. (C) Ramachandran plot determined by PROCHECK with 91.9% residues in the favored region of the plot.

The secondary structure of SS-MEVC consisted 35.61% of α-helices, 22.9% β-sheets, and 37.10% coils, indicating potential structural stability of SS-MEVC (S1 Fig). The high number of α-helices and coil-coiled domains in a vaccine construct is crucial for proper protein folding, mimicking native proteins structures, and confers sufficient and effective humoral immunity against the target pathogen [61]. The 3D-structure of SS-MEVC was predicted using the Robetta web tool and refined using the GalaxyRefine server. The first model, with an RMSD value of 0342 and a molProbity of 2.145 was chosen among the five refined structures for structural validation (Fig 3A and S2 Table). The quality score determined by ERRAT was 91.37 percent, whereas the Z-score was -4.78, according to ProSA-web (Fig 3B). A considerable number of the construct residues (91.9%) appeared in the favored region of the Ramachandran plot and ensured the 3D structural accuracy of the model construct (Fig 3C).

### Conformational B-cell epitope prediction

**Fig 4:**
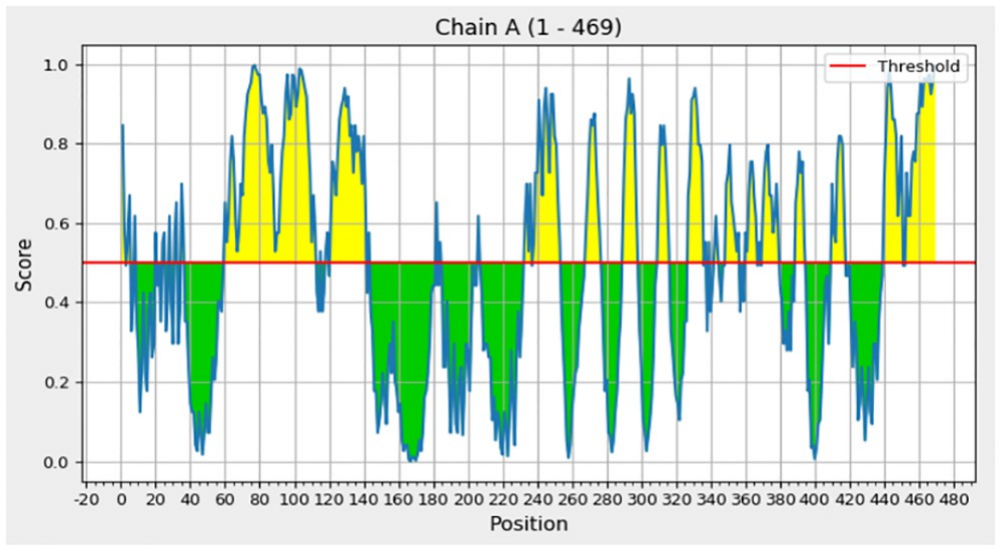
Residue-by-residue, two-dimensional ElliPro score charts for each conformational B-cell epitope in the SS-MEVC structure.

The refined structure of SS-MECV was submitted to ElliPro to determine the conformational B-cell epitopes. The server predicted a total of six discontinuous/conformational B-cell epitopes with scores ranging from 0.516 to 0.771 (S3 Table). Various lengths (138, 82, 12, 8, 4, and 4 residues) and compositions of the predicted conformational B-cell epitopes, A-F, were observed (S2 Fig and S3 Table). The scores for individual conformational B-cell epitopes from A-F are shown in Fig 4. These findings support a high prediction score for the six conformational B-cell epitopes in the SS-MEVC structure.

### Molecular docking of SS-MEVC with human immune receptors

**Fig 5:**
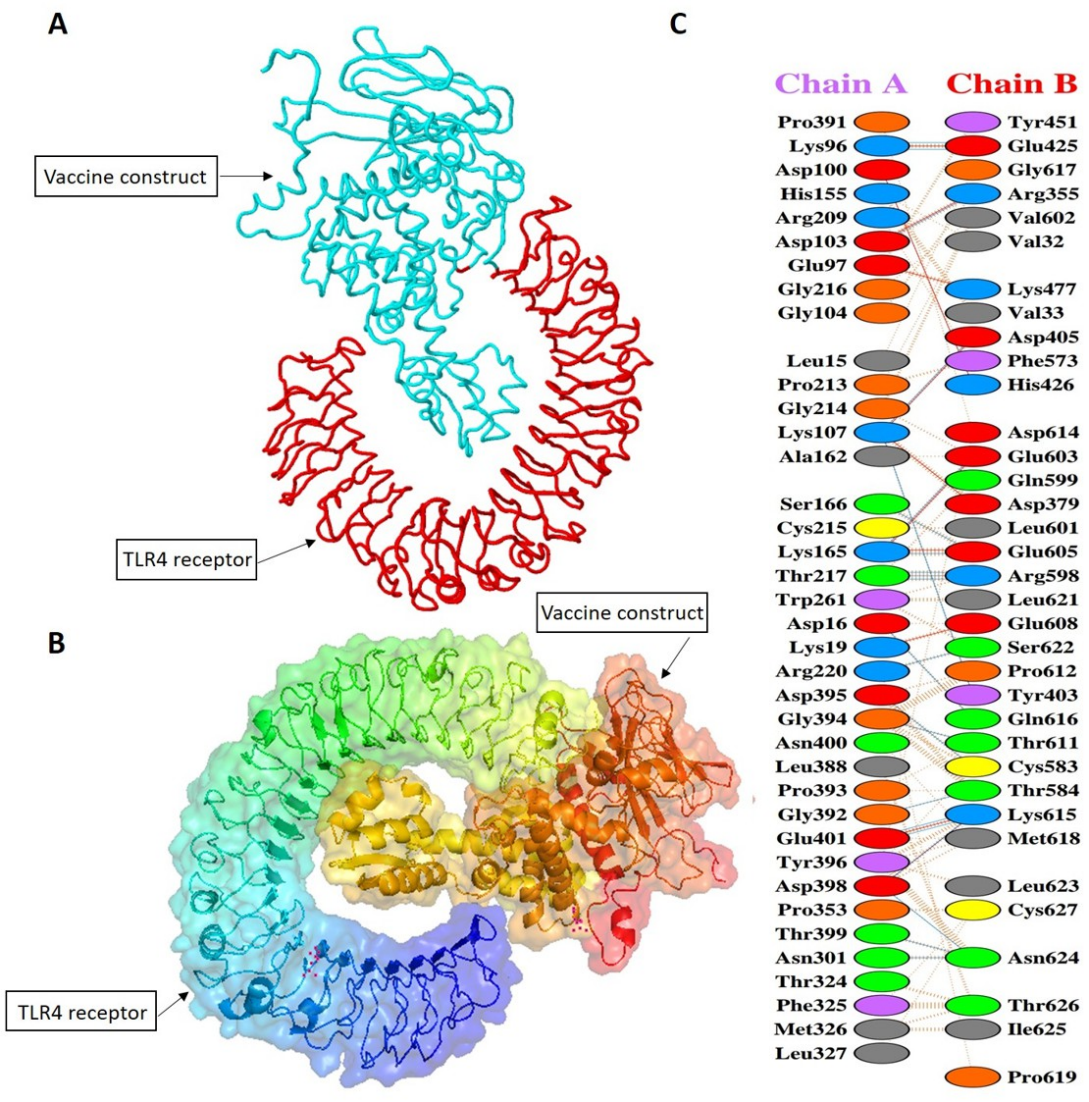
(A, B) 3D representation of the docking complex of SS-MEVC with human TLR4. (C) Molecular interactions between chain A of SS-MEVC and chain B of TLR4 molecules.

Molecular docking analysis was performed to evaluate the optimal binding conformation between the SS-MEVC and TLR immune receptors. The Cluspro server performed protein-protein rigid body docking of SS-MEVC with TLR2 and TLR4 based on millions of confirmations, RMSD-based clustering, and structural refinement based on energy minimization to generate the top 10 models. The complex model-4 was selected based on the lowest binding energies (-869.1 kcal/mol with TLR2 and -879.2 kcal/mol for TLR4) and high binding affinities for further analysis (Fig 5A and 5B and S3 Fig). Moreover, a graphical representation of the residues involved in the interactions between the top-ranked SS-MEVC-TLR4 docked complex (model-4) was generated using PDBsum (Fig 5C). A total of 22 hydrogen-bond interactions were observed between chain A of SS-MEVC and chain B of TLR4 molecules and 236 non-bonded contacts were also observed between SS-MEVC-TLR-4 chains.

### Normal modes analysis

**Fig 6:**
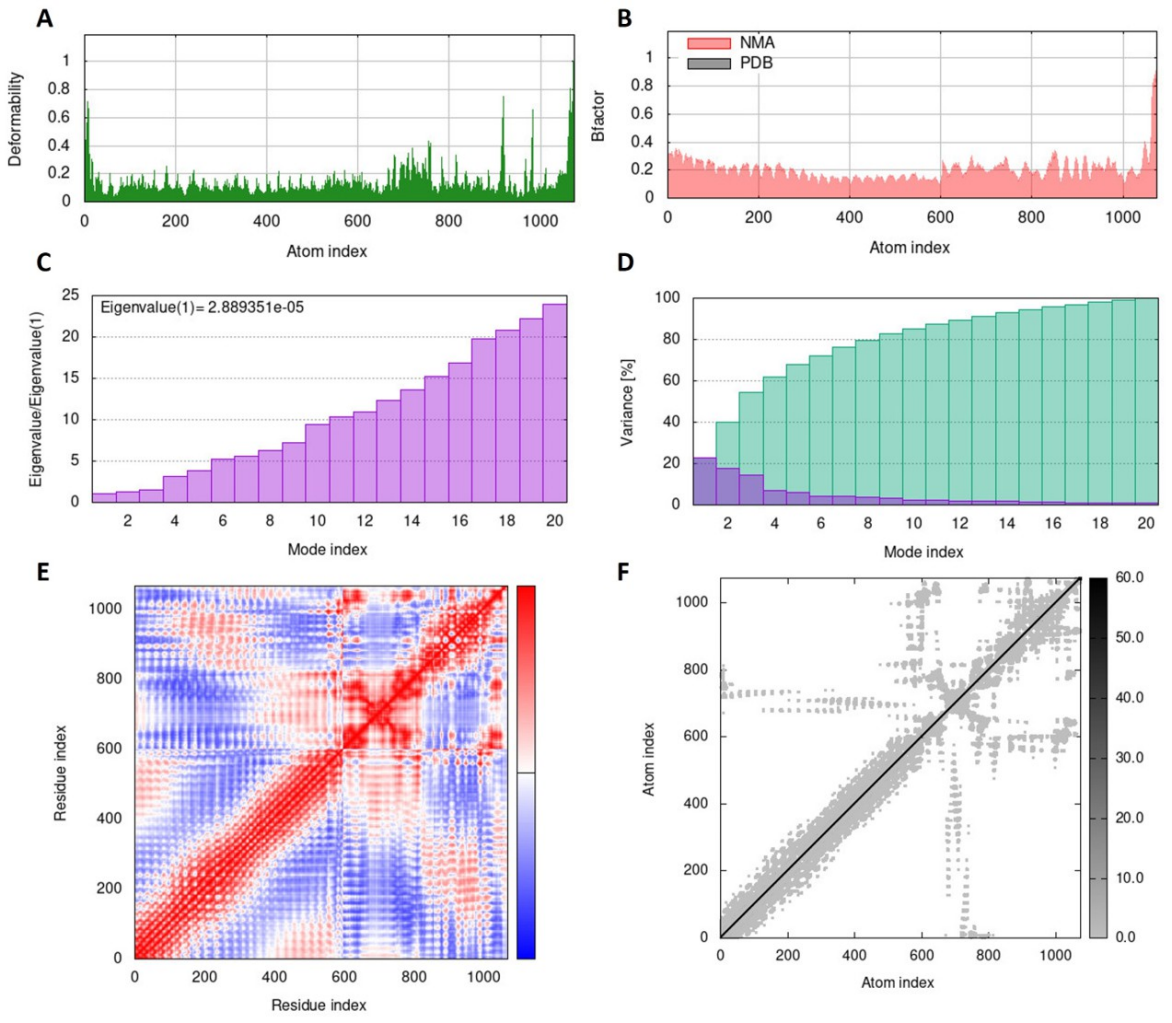
Results of normal mode analysis of vaccine SS-MEVC and TLR4 complex acquired by the iMODS server. (A) Deformability, (B) B-factor indicating average RMSD, (C) eigenvalue, and (D) colored bars showing the individual (purple) and cumulative (green) variances. (E) Covariance matrix indicating correlated (red), uncorrelated (white), and anti-correlated (blue) motions of paired residues and (F) the elastic network model of the SS-MEVC-TLR4 complex (grey regions indicating stiffer regions).

The collective functional mobility of SS-MEVC was determined using the iMODS web tool, which uses a normal modes analysis (NMA) approach to describe the functional motion of atoms in a macromolecule. Each normal mode has a frequency that correlates with the relative motion amplitude and deformation vector, indicating the direction of the atomic displacement of the macromolecule to assess its molecular flexibility in a cellular environment [62]. The NMA results for the SS-MEVC and TLR4 docked complex are shown in Fig 6. The RMSD of SS-MEVC-TLR4 complex was minimized by iterative deformation of the input structure along the lowest modes, while superimposing the two structures locally and globally to simulate potential transitions. A measure of the main-chain deformability is the total atomic displacements across all modes of residues at each individual atomic site. The peaks in the deformability graph of the complex indicate the flexible regions of the protein. The rigid regions of the main-chain residues showed lower values, whereas high values demonstrated flexible regions (hinges/linkers) of the chain (Fig 6A). The amplitudes of the atomic displacements of the molecular complex around the equilibrium conformation were determined using NMA-derived B-factor. The B-factor graph depicts the connection between NMA mobility of the SS-MEVC-TLR4 complex and the PDB scores, representing the average RMSD value (Fig 6B). The motion stiffness is represented by the eigenvalue associated with each normal mode, which is proportional to the amount of energy required to deform a structure. The lower the eigenvalue, the easier it is for α-carbon atoms to deform. The eigenvalue of the SS-MEVC-TLR4 complex was 2.889351e-05, indicating the significant stability of the complex (Fig 6C). Additionally, the variance graph was inversely related to the eigenvalue and associated with each normal mode of the SS-MEVC-TLR4 complex, representing individual (purple) and cumulative (green) variances (Fig 6D). The covariance map of the SS-MEVC-TLR4 complex revealed connectivity between pairs of residues in the system. The atomic movements in the dynamic regions of the complex molecule are depicted using covariance analysis as correlated (red), uncorrelated (white), or anti-correlated (blue) regions in the SS-MEVC-TLR4 complex (Fig 6E). *Ichiye and Karplus*, *1991* [63], calculated the correlation matrix using Cα Cartesian coordinates and Equation 2. The interactions between atoms were described by the elastic network model of the complex. Each dot in the graph denotes a spring connecting the corresponding pair of atoms. The stiffness of the dots is indicated by their color; darker greys denote stiffer parts, whereas the lighter dots denote more flexible sections (Fig 6F).

### Molecular dynamic simulations

**Fig 7:**
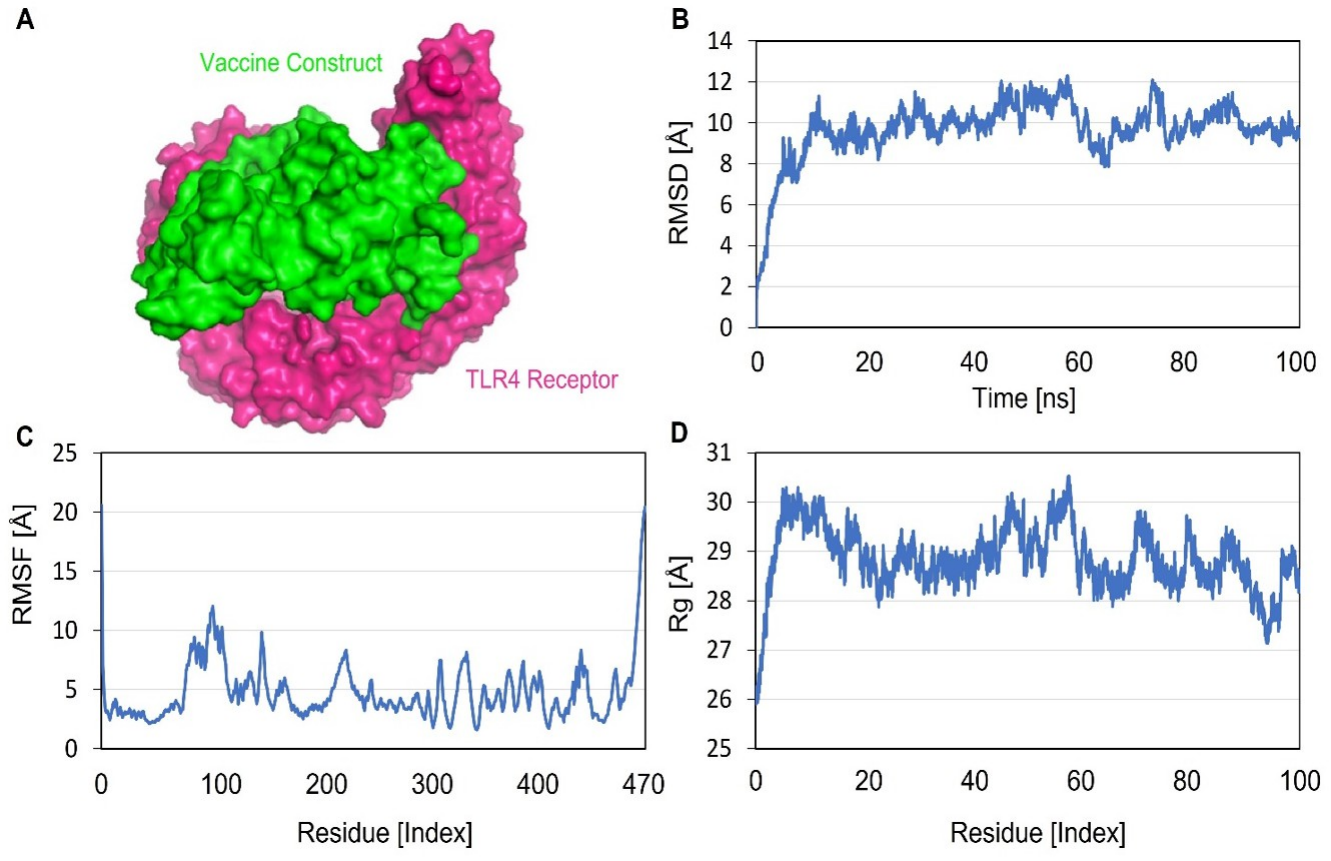
Molecular dynamic simulation of the SS-MEVC-TLR4 complex at 100ns using AMBER20. (A) Simulated the SS-MEVC-TLR4 docked complex, where the green color indicates vaccine construct and purple color indicates TLR4 receptor. (B) RMSD analysis of the SS-MEVC-TLR4 complex. (C) RMSF analysis of the SS-MEVC-TLR4 complex (D) Rg analysis of the SS-MEVC-TLR4 complex.

**Fig 8:**
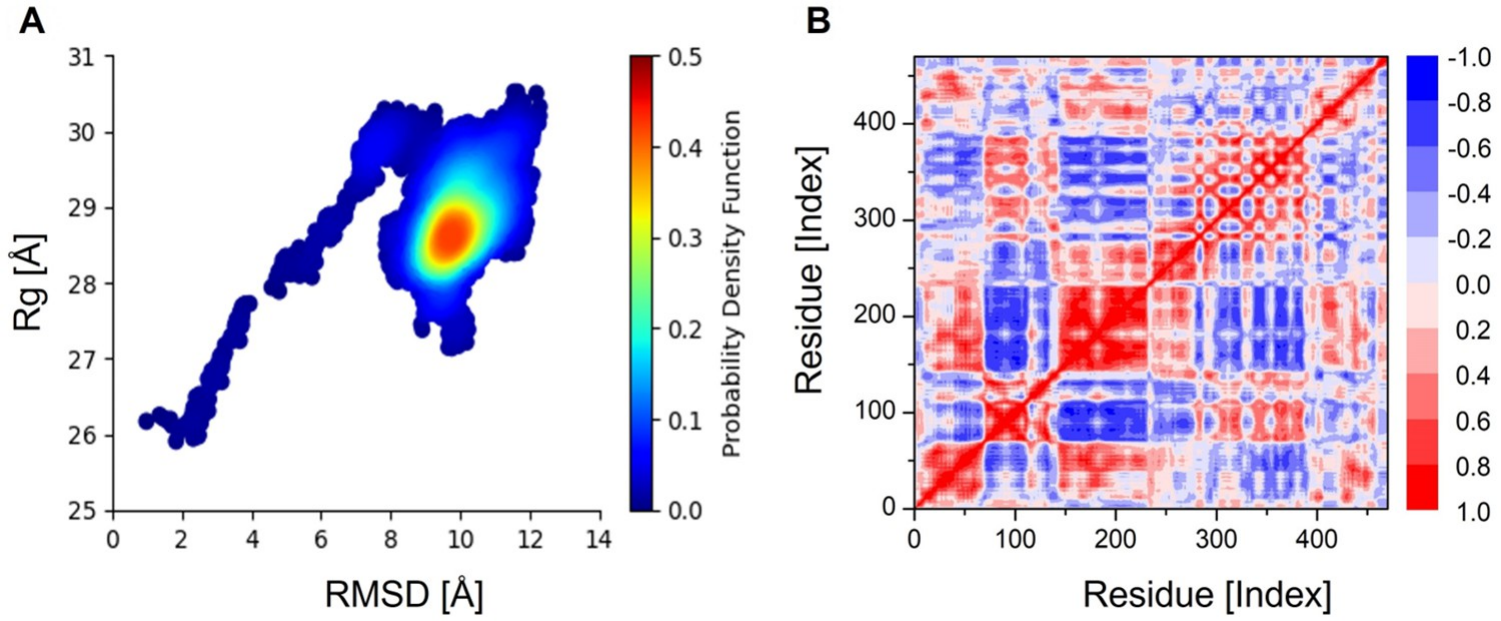
Probability distribution function and dynamic cross correlation matrix of SS-MEVC-TLR4 complex. (A) Demonstration of the distributions of RMSD and Rg to show the favored conformation as a contour plot. (B) Per-residue correlation matrix showing the correlated (red) and uncorrelated (blue) movements of SS-MEVC.

Computational molecular dynamic (MD) simulations analysis mainly evaluates the dynamic behavior of the docked molecules over a given period of time. MD simulations determine the correct binding conformations, protein folding, and stability in the cellular environment. An MD simulation of 100 ns was conducted to assess the dynamic behavior and structural stability of SS-MEVC. The trajectories of the MD simulation consisted of the RMSD, RMSF, and Rg analyses. The RMSD backbone atoms of the SS-MEVC-TLR4 docked complex revealed that the initial deviation was between 0.2–0.3 nm around 10 ns, however, RMSD plot was stabilized afterwards (Fig 7B). The convergence remained minimal and the average RMSD was observed to be 4.0 Å. RMSD analysis inferred stable molecular interactions between SS-MEVC and TLR4 docked complex. The flexibility of the backbone residues of the SS-MEVC-TLR4 complex was evaluated by RMSF analysis, showing lower fluctuation between SS-MEVC and TLR4 docked complex. However, the flexibility indices for the 80–120, and 288–300 regions were comparatively high, owing to the presence of loop regions. The residue fluctuation analysis revealed a lower flexibility index for the entire period of simulation of the overall system, and no drastic changes were observed in the SS-MEVC and TLR4 docked complex (Fig 7C). The Rg analysis for the SS-MEVC-TLR4 complex determined the compactness of the complex with slight variations, which depicted the statistical behavior of the vaccine in the system (Figs 7D and 8A). Moreover, the per-residue correlation matrix evaluated the correlated (red) and uncorrelated (blue) movements of the SS-MEVC vaccine (Fig 8B). Overall, MD simulation analysis indicated a stable binding affinity of the SS-MEVC vaccine with the TLR4 receptor, ensuring efficient representation of the vaccine to the host immune cells to elicit an immediate immunological response against the pathogen.

### Immune simulation

**Fig 9:**
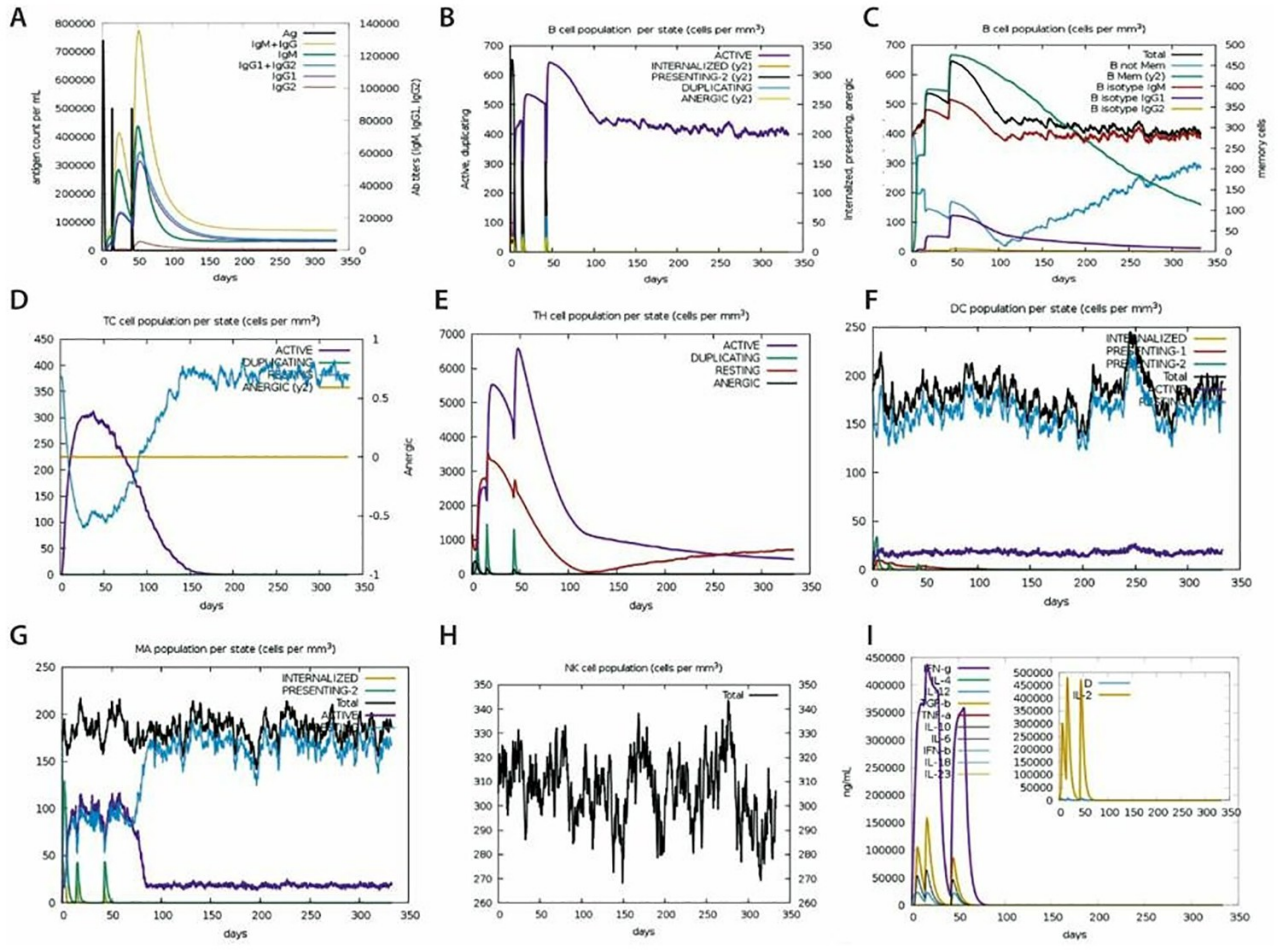
The *in silico* immune simulation of SS-MEVC vaccine peptide predicted by C-ImmSim server. (A, B) A significant increase in both B-cell numbers and antibody titer, with corresponding decreases in antigen concentrations. (C) An increase in B-cell numbers caused by repeated antigen exposure. (D, E) Increasing antigen exposure leads to an increase in T-cytotoxic and T-helper cells. (F, G, H) Increase in the number of dendritic cells, macrophages, and natural killer cell populations throughout the vaccination process. (I) Increase in the cytokine concentration after repeated antigen exposure.

Immune simulation predictions revealed a significant increase in primary and secondary immune responses induced by the proposed vaccine. The C-ImmSim online program was used to model a human immunization protocol utilizing SS-MEVC as an immunogen at three doses, one month apart. The simulation of immune responses to the antigen in a computer model accurately captured the complex dynamics of the immune system. Initially a significant increase in IgM levels was observed. The simulated secondary and tertiary responses revealed an increase in B-cell populations and IgG1 + IgG2, IgM, and IgM + IgG antibody levels associated with a decrease in antigen levels (Fig 9A and 9B). An increase in the memory B-cell population was also observed, indicating the formation of immunological long-term memories. An increase in both TC (cytotoxic) and TH (helper) cell populations in response to subsequent antigen exposure resulted in a drastic decline in antigen levels and the development of corresponding memory against the pathogen (Fig 9C–9E). Additionally, in this simulated immunization, the memory T helper cells and memory B cells were predicted to last for several months. Moreover, macrophages, dendritic cells, and natural killer cell populations were predicted to induced and maintained at high levels throughout the vaccination period (Fig 9F–9H). Similarly, significantly higher levels of cytokine-like IFN-γ and interleukin-like IL-2 were observed (Fig 9I). These results suggest that the predicted SS-MEVC could elicit sustainable and long-lasting humoral responses against *S*. *sonnei*.

### Condon optimization and *in silico* restriction cloning

Potent and stable antigenic SS-MEVC capable of eliciting both innate and adaptive immune responses must be produced using appropriate expression vectors. The JCAT server was used for codon optimization and reverse translation of the peptide SS-MEVC sequence into cDNA to achieve higher expression of the vaccine in the *E*. *coli* expression vector. The CAI value of SS-MEVC was predicted as 0.88, and the average GC content was 54.34%. JCAT server predicted an acceptable range for significant expression of SS-MEVC in the *E*. *coli* expression system. The codon sequence of SS-MEVC was inserted into the *E*. *coli* pET28a(+) sequence at Xbal (922) and MluI (2131) restriction sites to construct a recombinant plasmid sequence using the SnapGene tool. These results confirmed heterologous cloning and expression of SS-MEVC in the *E*. *coli* system (S4 Fig). The resulting cloned product could be used to transform the referred *E*. *coli* strain to produce the vaccine, following purification processes as per the immunization protocol.

## Discussion

*S*. *sonnei*, an *Escherichia coli* pathovar, is a gram-negative bacterium that causes bacillary dysentery and bloody diarrhea in humans. Currently, *S*. *sonnei* is a globally emerging pathogen and the leading cause of shigellosis in high-income countries. The unusually high number of cases of multi drug-resistant (MDR) and extensively drug-resistant (XDR) *S*. *sonnei* is a public health concern, as treatment options for moderate to severe cases are extremely limited [64]. Antibiotic resistance in bacteria has reached to a dangerous level, and strains resistant to most of the commonly used antibiotics are now routinely reported in many countries worldwide [65]. Therefore, new therapeutic strategies are needed to prevent MDR and XDR shigellosis. As antibiotic resistance increases, effective vaccine development has become the best alternative against such pathogens. Subtractive proteomics have been widely used to identify potential therapeutic targets for various pathogens. Advanced immunoinformatics and vaccinomics approaches are currently gaining interest in identifying potential pathogen-specific vaccine targets and designing potent vaccines against multiple resistant pathogens [66]. Reverse vaccinology and immunoinformatics approaches are cost- and time-effective with high efficacy compared to conventional vaccine development methods. The strategy utilized in the present study has been experimentally validated in mice models for various pathogens [67]. In 2019, researchers tested the ability of computationally generated multi-epitope vaccine to elicit a strong IgG antibody-specific immune response against *Acinetobacter baumannii* in mice [68]. Similarly, reverse vaccinology approaches have been experimentally validated in mice against *Salmonella Typhimurium* using an S. *Typhimurium* LT2 challenge model with FliK and BcsZ antigens [69]. The immunogenic responses of computationally designed B-cell epitopes to *Trypanosoma vivax* were experimentally validated [70]. Moreover, computational vaccines against several human viruses, such as Ebola [71] and Marburg [72], have been experimentally tested based on immunoinformatic strategies. This emphasizes the viability of *in silico* reverse vaccinology-based strategies for vaccine development.

Currently, no efficacious vaccines are available to prevent shigellosis. Previously, surface proteins of *S*. *sonnei* reference strain 53G were reported as potential vaccine candidates [73] and epitope mapping was performed [74]. However, the authors did not pursue detailed analyses to design and evaluate a proper multi-valent vaccine construct model against *S*. *sonnei*. Instead of single strain proteome data as utilized previously [74], we took advantage of the updated databases and analyzed relatively large data, i.e., comprised of multiple *S*. *sonnei* strains proteomes in the current study. Therefore, we speculate that the vaccine construct model prioritized in the current study may provide broader immune protection against the infection of multiple wide-spread strains of *S*. *sonnei*. Three proteins i.e., sulfur-transferase, putative oxidoreductase, and single-stranded DNA-binding protein were selected as top candidates based on immunological properties for designing a multimeric vaccine against *S*. *sonnei*. Immunogenic responses against infections are triggered by the host immune system via B- and T-cells. The prioritized proteins were used to predict B cell, MHC-I, and MHC-II epitopes to design a multi-epitope-based chimeric vaccine construct against *S*. *sonnei*. Various combinations of immune enhancers and flexible adjuvant peptides have been used to conjugate lead epitopes by using suitable epitope-specific linkers. Additionally, we examined the antigenicity, allergenicity, toxicity, solubility, and other physicochemical characteristics of the proposed vaccine construct. Vaccines designed from these pathogen-specific antigenic proteins have the capability to generate specific immunological responses, targeting conserved epitopes in the whole antigen while evading reactions to non-neutralizing epitopes that could lead to immunopathogenic or immune-modulating effects [75, 76].

Structural information is crucial for vaccine development to investigate interactions between antigen and receptor molecules. The 3D-structure of SS-MEVC was predicted based on homology modeling, and was further refined to improve. The structural stability of the refined 3D-structure of SS-MEVC was validated by the Z-score and Ramachandran plot, which determined the maximum number of residues in the favored region of the plot. Immunological and physicochemical properties determined the highly antigenic, non-allergenic, and non-toxic behavior of the SS-MEVC vaccine. Small molecular weight of~40 kDa, solubility, and instability scores ensured easy absorption, high soluble, and stable nature of the SS-MEVC vaccine upon expression in the host. Serological confirmation of immunoreactivity is an important step to validate the expression of the designed vaccine in a suitable expression system [77]. The *E*. *coli* expression vector is considered the most suitable expression system for cloning and development of recombinant peptides [78, 79]. The *in-silico* restriction cloning analysis inferred the successful cloning capability of the designed SS-MEVC vaccine within the bacterial expression system.

The immune simulation of the SS-MEVC vaccine was performed under different conditions to analyze the host immune responses. The analysis predicted a strong immune response induced by repeated antigen exposure. The consistently high level of Ig production and T cells, increasing levels of IFN-γ and IL-2 upon repeated antigen exposure predict strong cell-mediated and humoral responses against the designed SS-MEVC. The production of memory B cells indicated development of adaptive immunity that may last for several months to years. The Simpson index, D, for clonal specificity investigation indicated possible diverse immunogenic responses. Molecular docking analysis investigated the binding potential of SS-MEVC vaccine to the human TLR2 and TLR4 immune receptors. TLR receptors are crucial for immune cell activation to generate adaptive immune responses and thus play an important role in innate immunity [80]. TLR receptors recognize conserved bacteria-associated molecular patterns, leading to an intermolecular signaling cascade. including the activation of protein kinases, production of inflammatory cytokines, up regulation of MHC molecules, and activation of co-stimulatory responses. This links innate and adaptive immune responses, specifically T and B cells activation and memory responses when hosts encounter pathogens [81, 82]. The designed vaccine model was predicted at multiple-levels to potentially activate TLR receptors to initiate the innate immune response against *S*. *sonnei*. The stability of SS-MEVC and TLR4 docked complex was confirmed by MD simulation studies. Strong molecular interactions of the SS-MEVC vaccine with the TLR4 receptor ensured the molecular stability of the vaccine in the host cellular environment. These *in silico* analyses predicted that the chimeric vaccine model against shigellosis, proposed in current study, is extremely stable and capable of generating strong immunogenic responses. The next step is to conduct *in vitro* immunological assays to verify the efficacy of the proposed vaccine model constructs, assess its immunogenicity, and design a preclinical challenge-protection trial to validate the findings of this study.

## Conclusion

The present study employed an integrated subtractive proteomics and immunoinformatics approach to identify potential vaccine candidates against MDR *S*. *sonnai*. Highly antigenic B- and T-cell epitopes were prioritized to design a multi-epitope-based vaccine construct using suitable adjuvant and linker sequences to elicit the host immune system. Inmmunological and physiochemical properties ensured the antigenic, non-allergenic, non-toxic, and soluble behavior of the vaccine construct. Immune simulation predicted high cellular and humoral responses induced by the proposed vaccine with long-lasting innate immunity. Molecular docking ensured the binding affinity of the vaccine with human TLR4 receptor. MD simulations speculated the molecular stability of the vaccine in the cellular environment. *In silico* cloning predicted effective gene expression capability of the designed vaccine construct in *E*. *coli* expression system. Further *in vitro* and *in vivo* investigations are suggested to validate the efficacy of the proposed vaccine against *S*. *sonnai* infections.

## Supporting information

S1 File Multi-fasta file containing 2633 non-paralogous proteins from *S*. *sonnei* that were non-homologous to human acquired during subtractive proteomic analyses. (TXT)

S1 Fig The secondary structure of SS-MEVC predicted by PSIPRED server. The secondary structural elements are represented by the respective symbols. H shows the α-helices, β shows beta-sheets, and γ shows the coils in the secondary structure of SS-MEVC.
(TIF)

S2 Fig Predicted conformational B-cell epitopes in the SS-MEVC structure. According to the findings in Table 6, each field represents a single discontinuous B cell epitope (A–F).
(TIFF)

S3 Fig Molecular docking results of designed vaccine SS-MEVC with human TLR2 receptor. (TIF)

S4 Fig The *in silico* restriction cloning of SS-MEVC optimized codon sequence into the *E*. *coli* pET28a(+)expression plasmid. The sequence of the SS-MEVC is highlighted in red inserted between the restriction enzymes Xbal (922) and MluI (2131).
(TIF)

S1 Table CTL epitopes predicted by NetCTL 1.2 tool. (DOCX)

S2 Table The selection of an initial model for further analysis was based on obtained scores in comparison to the other five models generated by GalaxyRefine server. (DOCX)

S3 Table Predicted conformational B-cell epitopes in the SS-MEVC structure. (DOCX)

## Abbreviations

BLAST: Basic Local Alignment Search Tool
CAI: Codon Adaptation Index
CTL: Cytotoxic T Lymphocyte
GRAVY: Grand Average of Hydropathicity
HIV: Human immunodeficiency virus
HTL: Helper T Lymphocyte
IEDB: Immune Epitope Database
JCAT: Java Codon Adaptation Tool
LDMI: Developmental and Molecular Immunology
MD: Molecular Dynamic
MEVC: Multi-epitope Vaccine Construct
MHC-I: Major Histocompatibility Complex Class I
MHC-II: Major Histocompatibility Complex Class II
NCBI: National Center for Biotechnology Information
NICHHD: National Institute of Child Health and Human Development
NMA: Normal Mode Analysis
PDB: Protein Data Bank
Rg: Radius of Gyration
RMSD: Root-Mean-Square Deviation
RMSF: Root Mean Square Fluctuation
SMM: Stabilize Matrix Method
