# Supplementary figures and images for: Vaccinomics-aided next-generation novel multi-epitope-based vaccine engineering against multidrug resistant Shigella Sonnei: Immunoinformatics and chemoinformatics approaches
Source: PLoS One. 2023 Nov 22;18(11):e0289773. doi: 10.1371/journal.pone.0289773 (PMC10664945; doi:10.1371/journal.pone.0289773)

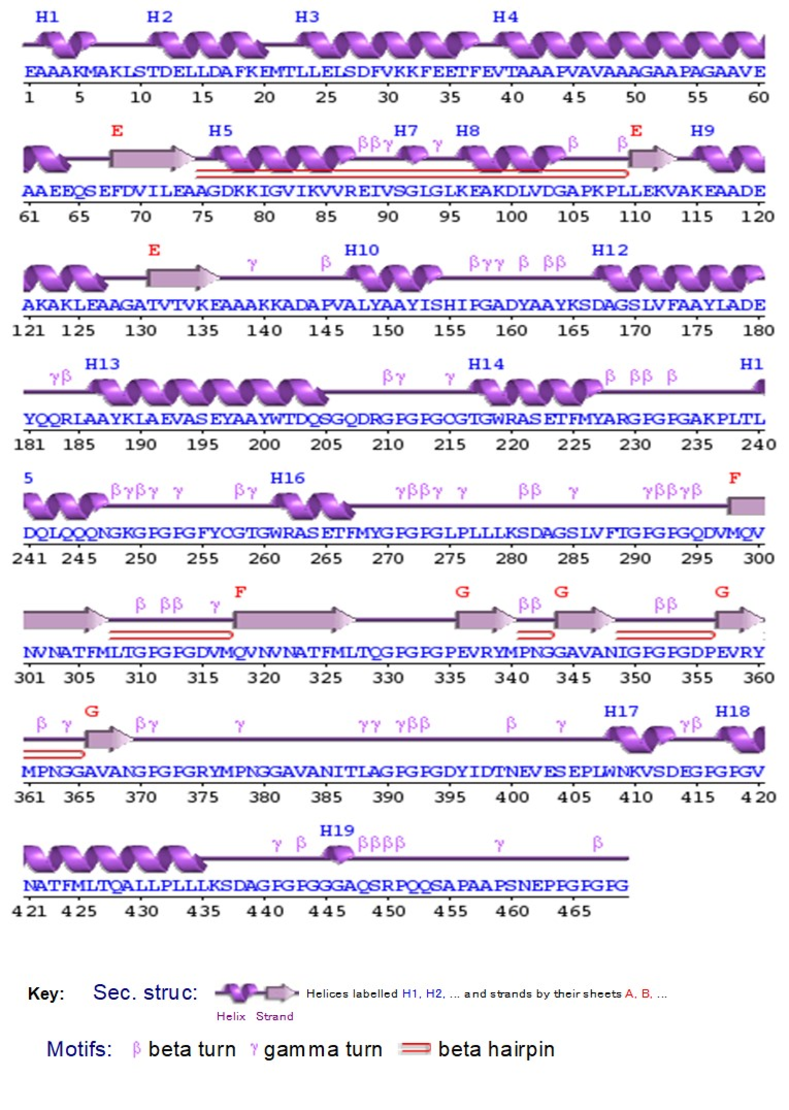

Supplement: S1 Fig — The secondary structural elements are represented by the respective symbols. H shows the α-helices, β shows beta-sheets, and γ shows the coils in the secondary structure of SS-MEVC. (TIF) [file pone.0289773.s002.tif]

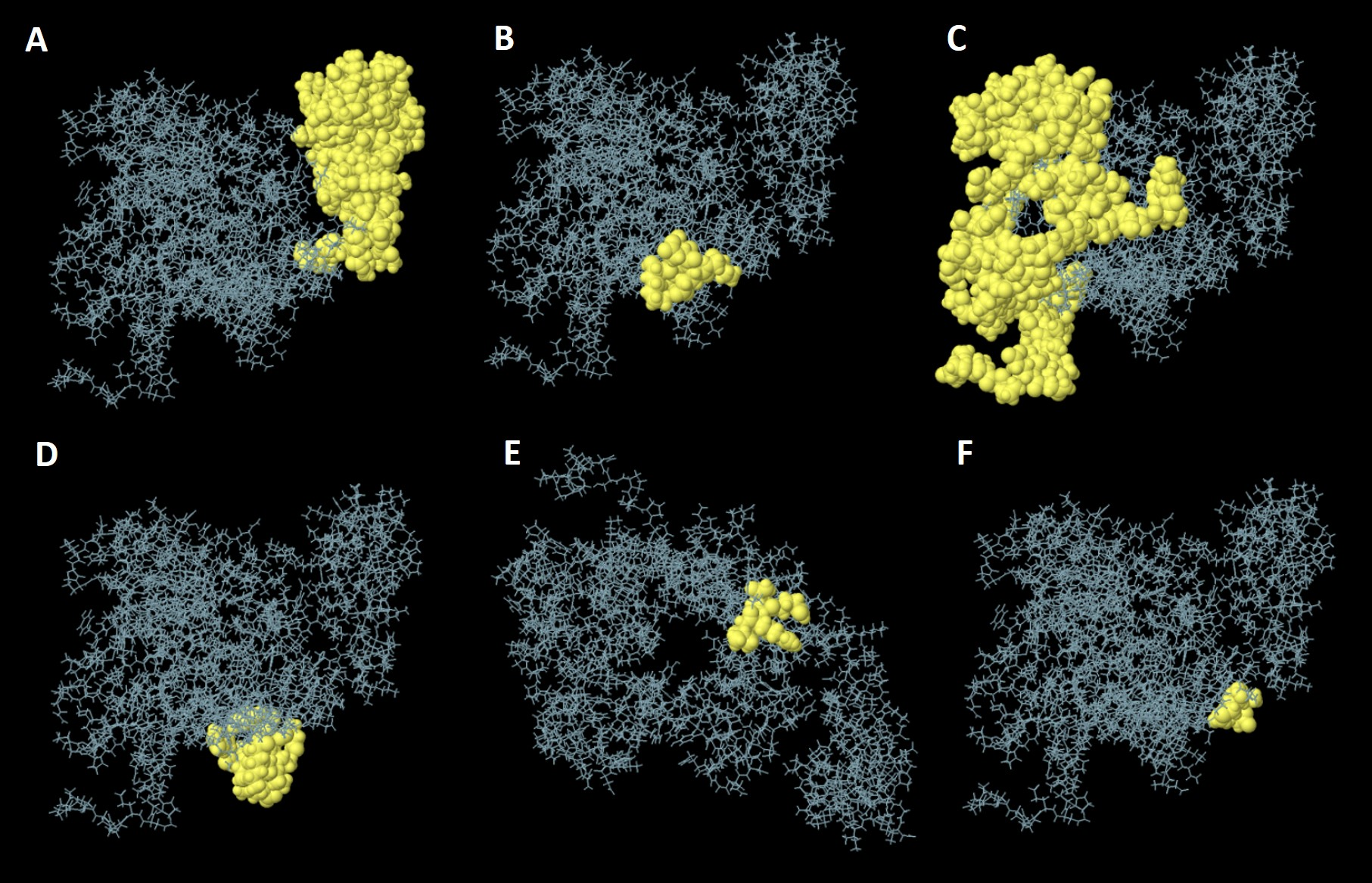

Supplement: S2 Fig — According to the findings in Table 6, each field represents a single discontinuous B cell epitope (A–F). (TIFF) [file pone.0289773.s003.tiff]

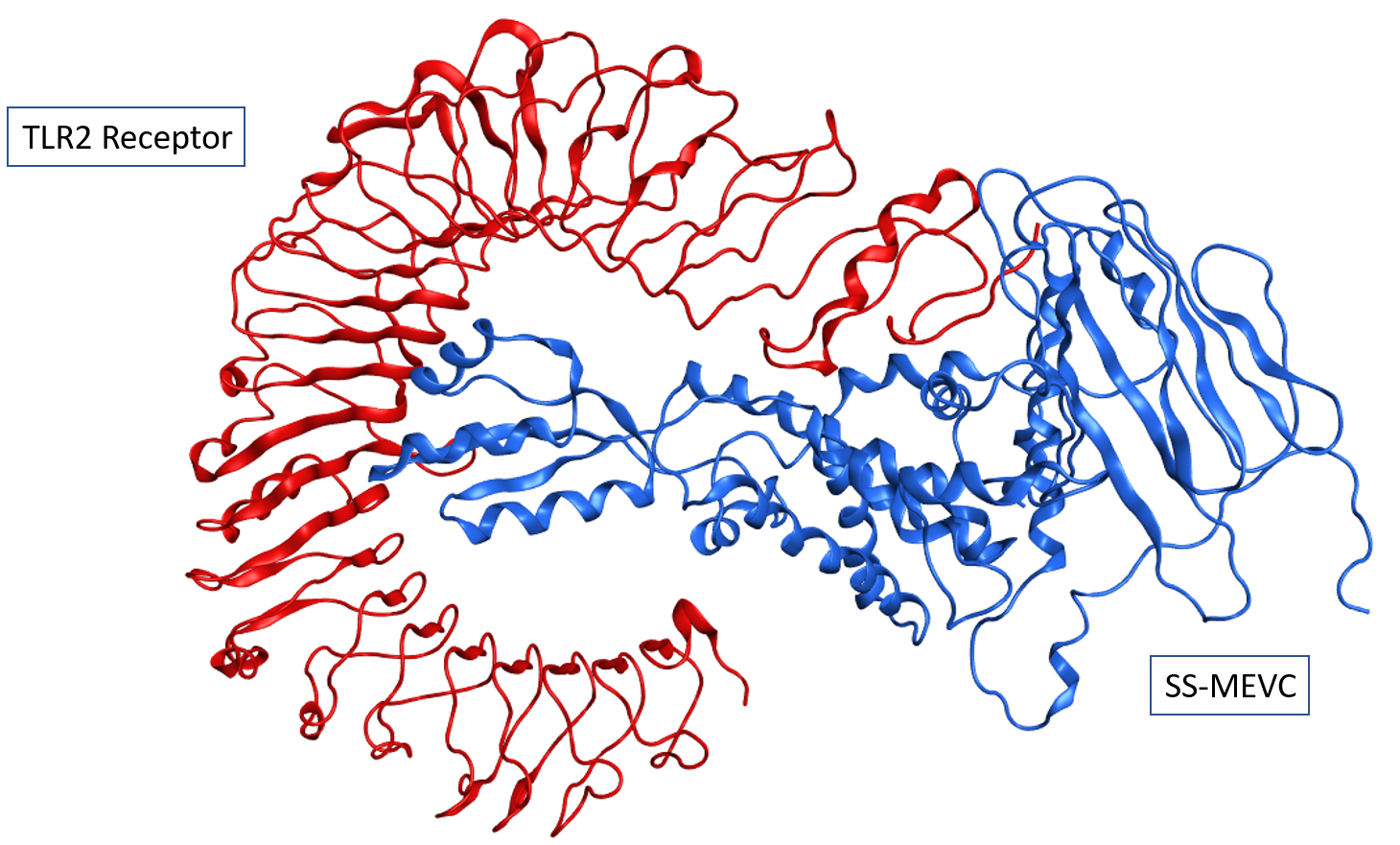

Supplement: S3 Fig — (TIF) [file pone.0289773.s004.tif]

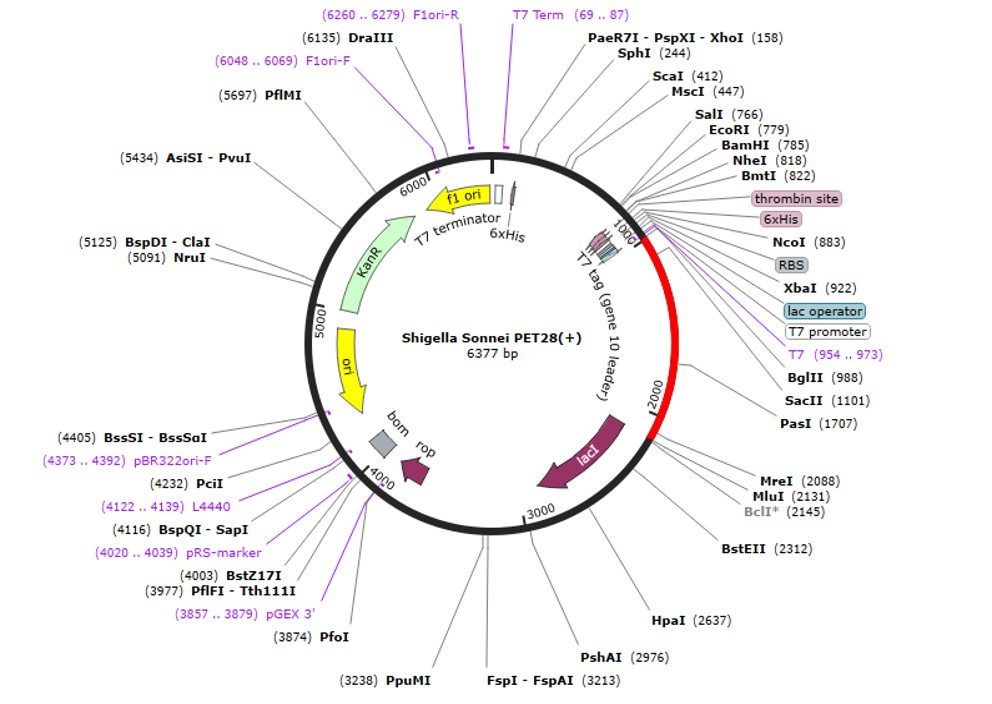

Supplement: S4 Fig — The sequence of the SS-MEVC is highlighted in red inserted between the restriction enzymes Xbal (922) and MluI (2131). (TIF) [file pone.0289773.s005.tif]
