# Supplementary material for: Vaccinomics-aided next-generation novel multi-epitope-based vaccine engineering against multidrug resistant Shigella Sonnei: Immunoinformatics and chemoinformatics approaches
Source: PLoS One. 2023 Nov 22;18(11):e0289773. doi: 10.1371/journal.pone.0289773 (PMC10664945; doi:10.1371/journal.pone.0289773)
Supplement: S1 Table — (DOCX) [file pone.0289773.s006.docx]

**Table S1:** CTL epitopes predicted by NetCTL 1.2 tool.

| **Residue No.** | **Peptide Sequence** | **MHC Bindig Affiniy** | **Resce Bindig Affiniy** | **C-Termil Cleave Affinity** | **Transport Affinity** | **Prediction Score** | **MHC-I Binding** | **Antigenecity Score** |
| --- | --- | --- | --- | --- | --- | --- | --- | --- |
| **99** | **KADAPVALY** | **0.4936** | **2.0959** | **0.9752** | **2.9560** | **2.3899** | **Yes** | **0.6672 Antigen** |
| 411 | VSVYDGGWY | 0.2644 | 1.1225 | 0.7005 | 3.2460 | 1.3899 | Yes | Non- Antigen |
| 226 | RHDTTVILY | 0.2418 | 1.0265 | 0.9695 | 3.0090 | 1.3224 | Yes | Non-Antigen |
| **188** | **ISHIPGADY** | **0.2002** | **0.8499** | **0.8738** | **3.0660** | **1.1343** | **Yes** | **0.5504 Antigen** |
| 394 | WRASETFMY | 0.1861 | 0.7900 | 0.9372 | 3.0910 | 1.0851 | Yes | Non-Antigen |
| 47 | AIDTRPSAF | 0.1629 | 0.6917 | 0.7216 | 2.7320 | 0.9366 | Yes | Non-Antigen |
| 85 | STEQLNAWI | 0.1885 | 0.8005 | 0.0958 | 0.4580 | 0.8378 | Yes | Non-Antigen |
| 367 | SADDITAW | 0.1339 | 0.5687 | 0.9760 | 0.9460 | 0.7624 | Yes | Non-Antigen |
| 175 | MMQVLADY | 0.4173 | 1.7717 | 0.7425 | 3.0310 | 2.0347 | Yes | Non- Antigen |
| **141** | **KSDAGSLVF** | **0.3999** | **1.6977** | **0.9411** | **2.5540** | **1.9666** | **Yes** | **0.4261 Antigen** |
| 27 | EAAMTYAY | 0.2331 | 0.9896 | 0.6939 | 2.9220 | 1.2397 | Yes | Non-Antigen |
| **82** | **LAQRIVVNY** | **0.1465** | **0.6220** | **0.7575** | **2.9000** | **0.8806** | **Yes** | **0.3677** **Antigen** |
| 198 | RTAMRASAF | 0.1654 | 0.7024 | 0.1613 | 2.7840 | 0.8658 | Yes | Non-Antigen |
| 179 | LADEYQQRL | 0.1368 | 0.5807 | 0.9674 | 0.8820 | 0.7699 | Yes | Non-Antigen |
| **63** | **KLAEVASEY** | **0.3174** | **1.3477** | **0.9733** | **3.0380** | **1.6456** | **YES** | **0.4234 Antigen** |
| **89** | **WTDQSGQR** | **0.2222** | **0.9432** | **0.0652** | **1.3610** | **1.0210** | **Yes** | **1.3576 Antigen** |
| 71 | YLRKGSQVY | 0.1322 | 0.5611 | 0.9698 | 2.8060 | 0.8469 | Yes | Non-Antigen |
