# Supplementary material for: Vaccinomics-aided next-generation novel multi-epitope-based vaccine engineering against multidrug resistant Shigella Sonnei: Immunoinformatics and chemoinformatics approaches
Source: PLoS One. 2023 Nov 22;18(11):e0289773. doi: 10.1371/journal.pone.0289773 (PMC10664945; doi:10.1371/journal.pone.0289773)
Supplement: S2 Table — (DOCX) [file pone.0289773.s007.docx]

**Table S2**: The selection of an initial model for further analysis was based on obtained scores in comparison to the other five models generated by GalaxyRefine server.

| **Model** | **GDT-HA** | **RMSD** | **MolProbity** | **Clash score** | **Poor rotamers** | **Rama favored** |
| --- | --- | --- | --- | --- | --- | --- |
| Initial | 1.0000 | 0.000 | 1.739 | 4.2 | 0.4 | 90.5 |
| MODEL 1 | 0.9756 | 0.342 | 2.145 | 14.2 | 0.7 | 92.0 |
| MODEL 2 | 0.9782 | 0.327 | 2.130 | 14.1 | 0.7 | 92.3 |
| MODEL 3 | 0.9730 | 0.357 | 2.143 | 14.2 | 1.1 | 92.8 |
| MODEL 4 | 0.9743 | 0.360 | 2.145 | 14.2 | 0.7 | 92.0 |
| MODEL 5 | 0.9763 | 0.344 | 2.132 | 12.3 | 1.1 | 91.5 |
