# Supplementary material for: Vaccinomics-aided next-generation novel multi-epitope-based vaccine engineering against multidrug resistant Shigella Sonnei: Immunoinformatics and chemoinformatics approaches
Source: PLoS One. 2023 Nov 22;18(11):e0289773. doi: 10.1371/journal.pone.0289773 (PMC10664945; doi:10.1371/journal.pone.0289773)
Supplement: S3 Table — (DOCX) [file pone.0289773.s008.docx]

**Table S3:** Predicted conformational B-cell epitopes in the SS-MEVC structure.

| **Serial No.** | **Residues** | **Total number of residues** | **Scores** |
| --- | --- | --- | --- |
| 1 | A:E60, A:A61, A:A62, A:E63, A:E64, A:Q65, A:S66, A:E67, A:F68, A:D69, A:V70, A:I71, A:L72, A:E73, A:A74, A:A75, A:G76, A:D77, A:K78, A:K79, A:I80, A:G81, A:V82, A:I83, A:K84, A:V85, A:V86, A:R87, A:E88, A:I89, A:V90, A:S91, A:G92, A:L93, A:G94, A:L95, A:K96, A:E97, A:A98, A:K99, A:D100, A:L101, A:V102, A:D103, A:G104, A:A105, A:P106, A:K107, A:P108, A:L109, A:L110, A:E111, A:K112, A:V113, A:A114, A:K115, A:A118, A:D119, A:A121, A:K122, A:A123, A:K124, A:L125, A:E126, A:A127, A:A128, A:G129, A:A130, A:T131, A:V132, A:T133, A:V134, A:K135, A:E136, A:A137, A:A138, A:A139, A:K140, A:K141, A:A142, A:D143, A:A144 | 82 | 0.771 |
| 2 | A:K410, A:V411, A:S412, A:D413, A:E414, A:G415, A:P416, A:G417 | 8 | 0.704 |
| 3 | A:R229, A:G232, A:P233, A:G234, A:A235, A:K236, A:P237, A:L238, A:T239, A:L240, A:D241, A:Q242, A:L243, A:Q244, A:Q245, A:Q246, A:N247, A:G248, A:K249, A:G250, A:P251, A:G252, A:P253, A:G254, A:F255, A:M268, A:G270, A:P271, A:G272, A:P273, A:G274, A:L275, A:P276, A:L277, A:F288, A:T289, A:G290, A:P291, A:G292, A:P293, A:G294, A:Q295, A:D296, A:V297, A:L308, A:T309, A:G310, A:P311, A:G312, A:P313, A:G314, A:D315, A:V316, A:M317, A:L327, A:T328, A:Q329, A:G330, A:P331, A:G332, A:P333, A:G334, A:P335, A:V337, A:Y339, A:M340, A:P341, A:N342, A:G343, A:G344, A:I349, A:G350, A:P351, A:G352, A:P353, A:G354, A:D355, A:P356, A:Y360, A:M361, A:P362, A:N363, A:G364, A:G365, A:A366, A:A368, A:G370, A:P371, A:G372, A:P373, A:G374, A:R375, A:Y376, A:M377, A:P378, A:N379, A:G380, A:G381, A:N385, A:T387, A:L388, A:A389, A:G390, A:P391, A:G392, A:P393, A:G394, A:D395, A:S437, A:A439, A:G440, A:P441, A:G442, A:P443, A:G444, A:G445, A:G446, A:A447, A:Q448, A:S449, A:R450, A:Q453, A:S454, A:A455, A:P456, A:A457, A:A458, A:P459, A:S460, A:N461, A:E462, A:P463, A:P464, A:G465, A:P466, A:G467, A:P468, A:G469 | 138 | 0.701 |
| 4 | A:E1, A:A2, A:A4, A:K5, A:K8, A:D28, A:K31, A:K32, A:E35, A:T36, A:F37, A:Y181 | 12 | 0.6 |
| 5 | A:Q182, A:Q183, A:R184, A:L185 | 4 | 0.529 |
| 6 | A:M21, A:T22, A:L24, A:E25 | 4 | 0.516 |
